# Design and Synthesis of Odorranalectin-Derived Peptides for RNA Binding

**DOI:** 10.21203/rs.3.rs-9282405/v1

**Published:** 2026-04-08

**Authors:** Tamara Damjanovic, Predrag Cudic

**Affiliations:** Florida Atlantic University; Florida Atlantic University

**Keywords:** Charged cyclic peptides, odorranalectin, RNA, binding, intranasal delivery

## Abstract

Small interfering RNA (siRNA) holds significant therapeutic potential for a broad range of diseases, including diseases of the central nervous system, yet its clinical translation remains limited by the lack of efficient and targeted delivery strategies, largely due to poor membrane permeability. To address this challenge, we developed a multifunctional RNA delivery platform by integrating positively charged cell-penetrating peptide sequences into the lectin-mimicking cyclic peptide odorranalectin (OL), which has demonstrated nose-to-brain transport capability. Placement of the cationic sequence at the *N*-terminus minimizes steric interference between functional domains, preserving the intrinsic carbohydrate-binding capability of the OL scaffold while enabling efficient RNA association. Peptides containing higher numbers of cationic residues exhibited enhanced RNA binding, as assessed by electrophoretic mobility shift assays and by increased thermal stability of peptide/RNA complexes measured using circular dichroism spectroscopy. Importantly, incorporation of the cationic sequence did not compromise carbohydrate recognition, as confirmed by isothermal titration calorimetry using OL-TAT and asialofetuin as a model system. Collectively, these results support the development of OL-based bifunctional ligands with tunable RNA and carbohydrate recognition and underscore their potential as intranasal platforms for targeted siRNA delivery to the brain.

## Introduction

Small interfering RNA (siRNA) molecules hold great promise as therapeutic agents for a wide range of diseases due to their ability to selectively silence disease-causing genes ^1–4^. siRNA-based therapies introduce synthetic siRNA into target cells to trigger RNA interference (RNAi), thereby suppressing specific messenger RNA (mRNA) and achieving gene silencing. The first commercial RNAi therapeutic, Onpattro (patisiran), was approved by the European Medicines Agency in 2018 for treating hereditary amyloidogenic transthyretin (hATTR) amyloidosis.^5^ This milestone paved the way for additional nucleic acid-based therapies, including Givlaari (givosiran, approved in 2019 for acute hepatic porphyria), Oxlumo (lumasiran, approved in 2020 for primary hyperoxaluria type 1), and Leqvio (inclisiran, approved in 2021 for atherosclerotic cardiovascular disease).^6–8^ Despite these successes, RNA-based drug development remains challenging due to delivery and stability obstacles.^9–11^

Brain and central nervous system (CNS) disorders are among the leading causes of disability worldwide, yet treatment is hindered because most therapeutic agents, including siRNA, cannot cross the blood–brain barrier (BBB) or blood–cerebrospinal fluid barrier (BCB).^2, 12–14^ Intranasal (i.n.) administration offers a promising alternative by enabling therapeutics to bypass these barriers and directly access the brain.^15–16^ Furthermore, i.n. delivery minimizes systemic exposure, reducing side effects and hepatic or renal clearance and toxicity.^17^

To develop a novel i.n. delivery platform for brain targeting, we designed a strategy based on a cyclic peptide scaffold with bioadhesive properties. We selected odorranelectin (OL), YASPK-*cyclo*(CFRYPNGVLAC)T, a naturally occurring 17-amino acid cyclic peptide, as the carrier. OL exhibits lectin-like binding, preferentially recognizing L-fucose and, to a lesser extent, D-galactose and *N*-acetyl-D-galactosamine, carbohydrates abundantly expressed on the olfactory epithelium of the nasal mucosa.^18–20^ This binding profile suggests that OL may prolong nasal residence time, enhancing drug absorption. To demonstrate OL’s utility for structural modification and i.n. delivery, we grafted a sequence of the potent μ-opioid receptor antagonist CTOP (f-*cyclo*(CYwOTX)T; X = penicillamine; O = ornithine) into the OL scaffold, generating a bicyclic peptide, OL-CTOP (YASPK-*cyclo*[CFRf-*cyclo*(CYwOTX)TC]T).^21^ Intranasal administration of OL-CTOP dose-dependently antagonized intracerebroventricular morphine-induced analgesia in mice and effectively prevented morphine-induced respiratory depression. These findings establish OL as a promising intranasal drug delivery vehicle and suggest that even large biomolecules, such as RNA, may be amenable to brain delivery via this route.

RNA-binding proteins (RBPs) play a critical role in gene regulation, influencing transcription, translation, trafficking, splicing, and decay.^22^ A common feature of RBPs is the arginine-rich motif (ARM), found in ribosomal proteins, lambdoid bacteriophage N proteins, and HIV-1 regulatory proteins Rev and TAT.^23–25^ Evidence suggests that ARMs can function as independent RNA-recognition domains. The multivalency of the guanidinium group enables simultaneous hydrogen bonding and stacking interactions with multiple RNA moieties, stabilizing protein/RNA complexes. Thus, it is not surprising that RBP inspired the design of peptide-based platforms for siRNA delivery.^26–30^ One notable example is a 29-mer peptide derived from rabies virus glycoprotein (RVG) conjugated to a nona-arginine (9R) sequence. This construct (RVG-9R) selectively targets nicotinic acetylcholine receptors on neurons, binds siRNA noncovalently, and enables transvascular transport into neurons, achieving gene silencing in mouse brain models.^28^ However, this strategy depends on the stability of the RNA/RVG-9R complex in systemic circulation and carries the risk of off-target systemic toxicity.

Inspired by the successful application of cell-penetrating peptides (CPPs) for RNA cellular internalization and building on our findings that OLs serve as an effective intranasal drug delivery platform,^21, 26–30^ we synthesized a series of OL derivatives incorporating polyarginine (Arg)_4,8,12_ and TAT_47–57_ (YGRKKRRQRRR) peptide sequences. These peptides were designed to explore the range of RNA-binding affinities. Their relative affinities for a model RNA were evaluated using electrophoretic mobility shift assays (EMSA) and by assessing the thermal stability of peptide/RNA complexes with circular dichroism (CD) spectroscopy. Additionally, the carbohydrate-binding capability of OL-TAT conjugate, relevant for potential nose-to-brain transport, was evaluated *via* isothermal titration calorimetry (ITC) using asialofetuin (ASF) as an olfactory glycoprotein model.

## Materials and Methods

### Chemicals and Reagents

TentaGel XV RAM resin was obtained from Rapp Polymer (Tuebingen, Germany). Fmoc-protected amino acids and coupling reagents (HOBt, HCTU) were purchased from Chem-Impex (Wood Dale, IL, USA) or Novabiochem (Gibbstown, NJ, USA). Triisopropyl silane (TIS), anisole and I_2_ were purchased from Sigma-Aldrich (St. Louis, MO, USA) and were of ACS reagent grade (> 99.8% purity). Trifluoroacetic acid (TFA) and methyl tert-butyl ether (MTBE) were purchased from Fisher Scientific (Atlanta, GA, USA) and were of ACS reagent grade (> 99.8% purity). Torula yeast (*Cyberlindnera jadinii*) was purchased from Sigma-Aldrich (St. Louis, MO, USA). Asialofetuin (ASF) was purchased from Sigma-Aldrich (St. Louis, MO, USA). HEPES sodium salt was purchased from Fisher Scientific (Atlanta, GA, USA). All solvents and other chemicals were purchased from Fisher Scientific (Atlanta, GA, USA) or Sigma-Aldrich (St. Louis, MO, USA) and were high-performance liquid chromatography (HPLC) grade.

#### Peptide Synthesis

All linear peptidyl-resin precursors were synthesized by Fmoc-SPPS on TentaGel XV RAM resin (substitution 0.2 mmol/g, 0.25 mmol scale) using an automated peptide synthesizer (Gyros Protein Technologies PS3 peptide synthesizer, Tucson, AZ, USA). Amino acid couplings were completed by using fourfold excess of amino acids and coupling reagents (HCTU/HOBt) in the presence of 0.4 M *N*-methylmorpholine (NMM) in dimethylformamide (DMF). Fmoc-deprotection cycles were carried out using 20% piperidine in DMF solution. Solid phase cyclization of OL and derivatives linear precursors *via* disulfide bond was carried out in a manual reaction vessel. In all cases, acetamidomethyl (Acm), a Cys sidechain protecting group, was removed *in situ* during the final cyclization steps (disulfide bridge formations) with I_2_ (2 eq), and anisole (4.4 eq) in DMF (10 mL) for 20 min. All peptides were cleaved from the resin, and all acid-sensitive sidechain-protecting groups were simultaneously removed using TFA/TIS/H_2_O (95:2.5:2.5, v/v/v). Peptides were precipitated with methyl tert-butyl ether, and the precipitate was collected with centrifugation, dissolved in water, and freeze-dried. Analytical RP-HPLC analyses and peptide purifications were performed on 1260 Infinity (Agilent Technologies, Santa Clara, CA, USA) liquid chromatography systems equipped with a UV/Vis detector. For analytical RP-HPLC analysis, a C18 monomeric column (Grace Vydac, 250 x 4.6 mm, 5 mm, 120 Å), 1 mL/min flow rate, and elution method with a linear gradient of 2 to 100% B over 45 min, where A is 0.1% TFA in H_2_O, and B is 0.08% TFA in CH_3_CN was used. For peptide purification, a preparative C18 monomeric column (Grace Vydac, 250 x 22 mm, 10 mm, 120 Å) was used. The elution method was identical to the analytical method except for the flow rate, which was 15 mL/min. MALDI-ToF mass spectrometry was performed on the Bruker Microflex LT system (Bruker, Billerica, MA, USA) in a reflector mode using an α-cyano-4-hydroxycinnamic acid matrix (positive-ion mode).

#### RNA purification and Characterization

Single-stranded ribosomal RNA from Torula yeast (*Cyberlindnera jadinii*) was purified by precipitation with 70% ice-cold ethanol [REF 49]. After purification, the RNA was mechanically sheared using a Fisher Scientific^™^ Model 505 Sonic Dismembrator at 25% power, applying five 20-second pulses to generate fragments approximately 600 bp in length.^31^ The average molecular weight per RNA base pair was used to estimate the molecular weight of the fragmented RNA to be ~ 160,000 g/mol, based on published values.^32^ RNA concentration and purity were determined by measuring UV absorbance at 260 nm following purification and fragmentation. The Torula yeast RNA used for gel electrophoresis and circular dichroism experiments was prepared at a concentration of 1.1 μM with an A260/280 ratio of 2.14, indicating high purity.

#### Gel Electrophoresis Mobility Shift Assay (EMSA)

The EMSA assay was used to evaluate binding between a constant RNA concentration (100 nM) and OL, OL–CPP, or linear cationic peptides. Peptide concentrations were varied to obtain molar ratios ranging from 0 to 100 relative to RNA. Each peptide sample was mixed with RNA and incubated for 5–10 minutes to allow complex formation. Following incubation, 10% glycerol was added to facilitate uniform gel loading. EMSA was performed on a 0.8% agarose gel using 0.5X TBE as the running buffer. A 5X TBE loading buffer was loaded into one lane as a mobility reference, as it contains bromophenol blue and xylene cyanol dyes that migrate at ~ 100–300 bp and 4–5 kbp, respectively. Electrophoresis was conducted for 20 minutes at 100 V. After separation, the gel was stained with SYBR Safe DNA Gel Stain for 15 minutes to visualize RNA bands and imaged using Image Studio.

#### Thermal Stability Analysis of Peptide/RNA Complexes by Circular Dichroism Spectroscopy

CD measurements were conducted in 10 mM Tris–HCl buffer (pH 7.5) using a Jasco J-810 spectropolarimeter (Jasco, Easton, MD) equipped with a 1 mm path-length quartz cuvette. Temperature-dependent CD analyses were performed from 10°C to 90°C at a fixed wavelength of 260 nm. The RNA concentration was maintained at 1.1 μM. Peptide concentrations were as follows: OL-(Arg)_4_, 37.1 μM; OL-(Arg)_8_, 19.5 μM; OL-(Arg)_12_, 11.5 μM; (Arg)_4_, 535.5 μM; (Arg)_8_, 37 μM; (Arg)_12_, 23.3 μM; TAT, 23.5 μM; OL-TAT, 17.9 μM; and OL, 14 μM. These concentrations correspond to approximately complete saturation of the RNA as determined by EMSA. Each sample was analyzed in triplicate using the instrument’s variable-temperature mode. Spectra were processed in Spectragryph using the Savitzky–Golay algorithm for smoothing. Melting temperatures (T_m_) were determined from the inflection points of first-derivative plots of the temperature-dependent CD curves.

#### Isothermal Titration Calorimetry (ITC) Experiments

A binding study was performed at 25°C in 20 mM HEPES at pH 7.0 by using a titration calorimeter PEAQ-ITC (Malvern, Northampton, MA) with a reaction cell volume of 300 μL. Typically, ASF solutions (250 μM) were in the reaction cell and titrated with solutions of OL-TAT and TAT at a concentrations of 3.5 and 3 mM, respectively. OL-TAT and ASF were dialyzed and prepared in the same buffer. At least 18 consecutive injections of 2 μL were applied every 120 s interval at a constant stir speed of 750 rpm. The concentrations of OL-TAT and ASF were confirmed by measurements using a BioTek Epoch microplate spectrophotometer (Agilent Technologies, Santa Clara, CA, USA). The raw integrated heat plots were analyzed using the MicroCal PEAQ-ITC software v1.21 (Malvern) under the 1-set-of-sites model, and the control parameter as a fitted offset was applied to each titration as per the manufacturer’s guidelines and our previous applications.^20^ The reported thermodynamic parameters were derived from three independent experiments and then averaged.

## Results

### Design and Synthesis of OL-CPPs for RNA binding

The linear peptidyl–resin precursors for OL, OL-CPP, and CPP controls were synthesized by standard Fmoc-SPPS using low-substitution TentaGel XV RAM resin (0.2 mmol/g), Scheme 1. Our previous studies identified Lys_5_, Phe_7_, Tyr_9_, Gly_12_, Leu_14_, and Thr_17_ within the native OL sequence as critical for carbohydrate binding.^20^ Accordingly, the positively charged sequence was strategically positioned at the OL *N*-terminus to minimize steric interference that could otherwise compromise both carbohydrate and RNA binding.

The high swelling capacity and large effective reaction volume of this resin provide pseudo-dilution conditions that facilitate efficient on-resin cyclization and favor intramolecular disulfide bond formation.^33^ Disulfide formation was achieved by iodine-mediated oxidation, a rapid and selective method for converting Cys(Acm) residues to disulfides while remaining compatible with common Fmoc-SPPS protecting groups.^21, 34–35^ In all cases, oxidation was complete within 20 min, as confirmed by analytical RP-HPLC and MALDI-TOF MS. The overall isolated yields of purified cyclic peptides ranged from 11–65%, depending on the number of arginine residues in the sequence. The lowest yield (11%) was obtained for OL-(Arg)_12_, whereas OL-(Arg)_4_ afforded the highest yield (63%). Similarly, for linear polyArg control peptides, (Arg)_12_ gave the lowest yield (7%) and (Arg)_4_ the highest (17%). These yield variations likely reflect both the challenges inherent to iodine-mediated Cys oxidation and the synthetic difficulties associated with incorporating multiple arginine residues during solid-phase peptide assembly.

### Assessment of Peptide/RNA Interactions by Electrophoresis Mobility Shift Assay (EMSA)

**Figure 1:**
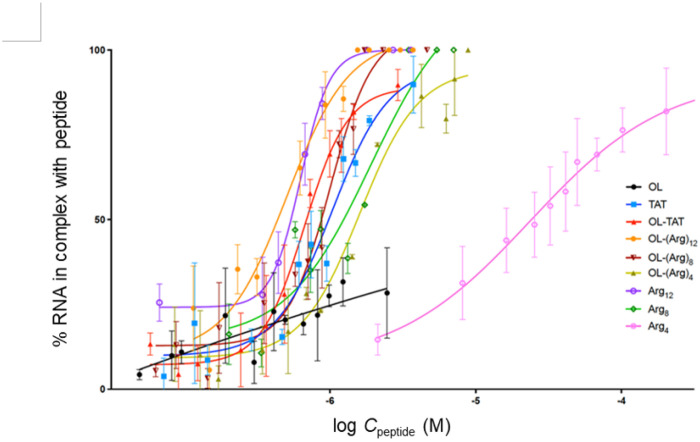
Assessment of Peptide/RNA interactions by gel electrophoretic mobility shift assay. Data are presented as % RNA bound to peptide (mean ± SD, n = 3) and fitted with a sigmoidal dose–response curve. The band intensity representing free RNA was quantified using ImageJ software.

The gel electrophoretic mobility shift assay (EMSA) was used to assess the interactions of OL, OL-CPP and linear CPP peptides with RNA. This assay monitors peptide/RNA by detecting decreases in electrophoretic mobility that result from complex formation, which produces slower-migrating or gel-retained species due to increased molecular size and altered charge.^36^ In all cases examined, peptide–RNA complexes remained at the gel origin, whereas unbound RNA migrated into the gel. The amount of free RNA was quantified using ImageJ software.^37–38^ Representative EMSA results are shown in Fig. 1.

Overall, the data demonstrate that the RNA-binding affinities of both OL-CPP and linear CPP peptides corelate with peptide charge; more positively charged peptides exhibit stronger RNA binding.

Comparisons between OL-CPP peptides and their linear CPP counterparts revealed no detrimental effect of the OL scaffold on RNA binding, Fig. 1. OL-(Arg)_12_ and linear (Arg)_12_ displayed identical and very strong affinities toward RNA. For all other peptides, however, notable differences in affinity were observed between OL-CPP peptides and their linear CPP counterparts, with the greatest difference observed between OL-(Arg)_4_ and (Arg)_4_, Fig. 1. The control OL peptide also showed also weak but detectable interaction with RNA, consistent with the presence of Arg and Lys residues in its sequence that can mediate electrostatic interactions with RNA.

### Assessment of the Thermal Stability of Peptide/RNA Complexes Using CD Spectroscopy

The thermal stability of OL-CPP and CPP peptide/RNA complexes was evaluated using circular dichroism (CD) spectroscopy by monitoring temperature-dependent changes in the CD signal.^39^ Melting temperatures (T_m_) were extracted from the minima of the first-derivative curves. A rightward shift in these minima reflects increased thermal stability of the peptide–RNA complexes, consistent with stabilization of RNA secondary structure upon peptide binding. Melting temperatures for all complexes are summarized in Table 1.

In agreement with the EMSA results, OL-(Arg)_12_ produced the greatest enhancement in RNA thermal stability, exhibiting a Tm of approximately 43°C, substantially higher than that observed for the other peptide conjugates. OL-(Arg)_12_ was followed in stabilizing efficacy by OL-TAT, OL-(Arg)_8_, and OL-(Arg)_4_. A similar trend was observed among the linear cationic peptides, where increasing numbers of arginine residues correlated with increased RNA stabilization. Unexpectedly, both the linear (Arg)_4_ peptide and OL alone, despite exhibiting relatively weak RNA binding in EMSA, conferred a stronger stabilizing effect on RNA than OL-(Arg)_4_ as well as the OL-TAT and TAT peptides.

### Characterization of OL-TAT/Glycoprotein and TAT/Glycoprotein Interactions by ITC

Our previous studies demonstrated that OL binds to ASF, a model glycoprotein bearing terminal Gal and GalNAc residues, with μM affinity, and that its cellular uptake likely occurs *via* a receptor-mediated transcytosis mechanism.^20, 40^ Therefore, it is critical that OL–CPP peptides retain glycoprotein-binding capability to facilitate efficient nose-to-brain transit and cellular uptake. We hypothesize that strategic placement of positively charged sequences at the OL scaffold *N*-terminus will allow RNA binding while preserving OL’s carbohydrate-binding function and minimizing steric hindrance from the OL core and CPP chains.

**Figure 2:**
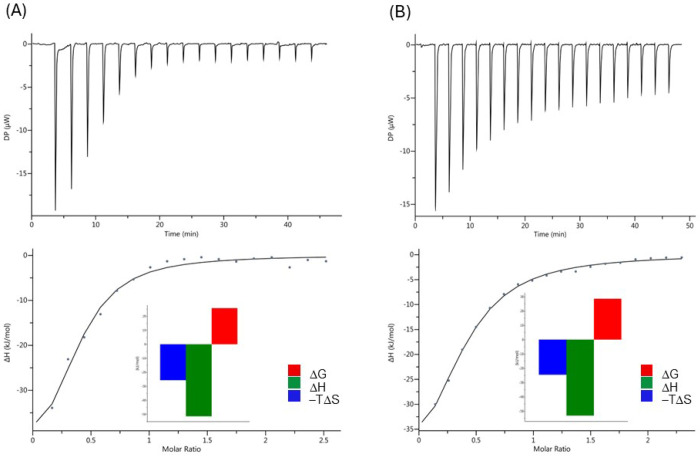
ITC titration profile of (A) ASF (250 μM) with OL-TAT (3.5 mM), and (B) ASF (250 μM) with TAT (3 mM), in 20 mM HEPES buffer (pH 7.0). The resulting values for stoichiometry (n = 0.3), dissociation constants (OL-TAT *K*_d_= 32.5 mM and TAT *K*_d_ = 51.6 mM), ΔH (OL-TAT = - 25.65 kJ/mol and TAT = - 24.48 kJ/mol), ΔG (OL-TAT = - 51.46 kJ/mol and TAT = - 53.14 kJ/mol), -TΔS (OL-TAT = 25.73 kJ/mol and TAT = 28.54 kJ/mol) were obtained using the one-set-of-sites model in the MicroCal PEAQ-ITC analysis software v1.21.

To evaluate whether OL–CPP peptides can interact with relevant carbohydrates, we conducted a binding study using ASF and OL–TAT. OL–TAT was selected because the TAT peptide is a well-characterized RNA delivery platform widely described in the literature.^29–30, 41^ Binding affinities of OL–TAT and TAT peptides toward ASF were determined by isothermal titration calorimetry (ITC). As shown in Fig. 2, OL–TAT binds ASF with a dissociation constant (*K*_d_) of 32.5 μM and the TAT peptide binds ASF with a *K*_d_ of 51.6 μM. The observed TAT/ASF binding may stem from nonspecific electrostatic effects. The *K*_d_ value for OL–TAT is comparable to our previously reported OL/ASF binding affinity.^19–20^ The enthalpy change (ΔH) for OL–TAT binding to ASF was − 25.6 kJ/mol, while the entropy contribution (–TΔS) was 25.7 kJ/mol, indicating that the interaction is spontaneous, as both factors favor complex formation. These findings suggest that OL–TAT may bind carbohydrates expressed on the olfactory epithelium, potentially extending its residence time in the nasal cavity and enhancing brain uptake.

## Discussion

siRNA offers significant therapeutic potential due to its precise ability to silence pathogenic genes.^4^ However, efficient and targeted delivery of siRNA remains a major challenge. To address this, we developed a novel RNA delivery platform that integrates positively charged CPP sequences into a lectin-mimicking cyclic peptide OL. CPPs are known to bind oligonucleotides through electrostatic interactions, whereas OL has been shown to facilitate intranasal delivery of peptides and nanoparticles into the brain of mouse models.^21, 42^ We therefore hypothesized that positioning the CPP sequence at the OL *N*-terminus would reduce steric interference between the two functional domains, enabling RNA binding without compromising OL’s carbohydrate binding capability, Scheme 1.

Arginine is widely recognized as the most effective basic residue for enhancing oligonucleotide binding and complex stability.^25–26, 30^ This characteristic of arginine is attributed to unique physicochemical and structural features of its side chain. The guanidinium group of arginine has a higher p*K*_a_ than the primary amine of lysine, ensuring that it remains fully protonated across a wide physiological pH range and thereby enabling strong electrostatic interactions with the negatively charged phosphate backbone of nucleic acids. In addition, the planar guanidinium moiety also supports multivalent binding, allowing a single arginine residue to simultaneously engage in hydrogen bonding and stacking interactions with both the phosphate backbone and nucleobases, thereby strengthening overall complex stability.^43–45^

However, the solid-phase synthesis of CPP containing poly-arginine sequences is challenging. The cumulative steric bulk of guanidinium protecting groups (e.g., Pbf) reduces acylation efficiency, while the high local charge density and restricted chain mobility hinder resin swelling and solvent accessibility.^46–47^ These effects promote on-resin aggregation, incomplete Fmoc deprotection, and formation of truncated or heterogeneous products. Arginine rich peptides are also prone to side reactions during acidolytic cleavage, and their strong cationic character complicates RP HPLC purification by producing broad, strongly retained peaks.^47^ To minimize the steric bulk effect of guanidinium protecting groups and to ensure efficient on-resin cyclization of our OL-CPP constructs, we employed a high-swelling resin that provides an expanded reaction volume and pseudo-dilution conditions favorable for intramolecular disulfide bond formation during iodine mediated cysteine oxidation.^35^ As previously demonstrated, iodine promotes rapid, high yield oxidation while simultaneously removing S-acetamidomethyl (Acm) protecting groups, enabling deprotection and cyclization to occur in a single step.^21, 34^ The overall isolated yields of both linear and cyclic peptides decreased as the number of arginine residues increased. These yield trends likely reflect the combined challenges of iodine mediated Cys oxidation, including potential nonselective oxidation of tyrosine or over oxidation of cysteine to higher oxidation states, and the well-established synthetic difficulties associated with incorporating multiple arginine residues during solid phase peptide assembly.^34^

To investigate RNA binding by the OL-derived peptides, we performed Electrophoretic Mobility Shift Assays (EMSAs) using principles commonly applied to protein–nucleic acid complex analysis. EMSA is based on the observation that peptide/protein–RNA complexes migrate more slowly during electrophoresis than free RNA.^48^ As a result, RNA–peptide interactions produce characteristic mobility “shifts,” enabling qualitative assessment of complex formation as well as comparative evaluation of binding strength and specificity. In the resulting gels, the faster-migrating bands correspond to unbound RNA, while the slower or non-migrating bands represent peptide/RNA complexes. The intensity of the fast-migrating (free RNA) bands, quantified using ImageJ, is proportional to the concentration of unbound RNA and is used to estimate binding affinities.

Based on our EMSA results, the strongest RNA binding was observed for highly cationic peptides, such as OL-(Arg)_12_, whereas OL-(Arg)_4_ showed the weakest binding, Fig. 1. Similar trends were observed for the linear CPP control peptides. Interestingly, the data also suggest that in weaker RNA binders, additional stabilization of RNA-peptide complexes may arise from interactions involving the OL ring itself. This effect could be explained by the presence of positively charged Lys and Arg residues within the OL sequence, which may interact with the negatively charged phosphate groups of RNA.

Identical trends were observed in the circular dichroism (CD) spectroscopy analysis of the thermal stability of peptide/RNA complexes, Table 1. Strong RNA binders, such as OL-(Arg)_12_ and (Arg)_12_, formed more stable complexes with RNA, resulting in increased melting temperatures (T_m_). However, the stabilizing influence of the OL ring on peptide/RNA complex formation appeared less pronounced in CD measurements compared with the EMSA results. Notably, the parent OL exhibited a substantial stabilizing effect on its RNA complex, an effect not detected by EMSA. This discrepancy likely reflects differences in the sensitivities of the two methods. CD thermal unfolding experiments are highly sensitive to shifts in population equilibria and do not directly reflect on binding strength.^49–52^ Even weak interactions that slightly favor the folded RNA state can produce measurable shift in T_m_. Thus, CD can reveal subtle stabilizing contributions that EMSA might not detect.

For efficient nose-to-brain transport of OL-CPP peptides, it is highly desirable that these peptides exhibit affinity toward carbohydrates expressed on the olfactory epithelium of the nasal mucosa. We have previously shown that OL-based peptides bind to model glycoproteins containing terminal Fuc, Gal, and GalNAc sugar moieties with micromolar affinities, and that some of these peptides can elicit biological responses in mice following intranasal administration.^20–21^ To determine whether OL derivatives modified at the *N*-terminal site by incorporation of positively charged CPP amino acid sequences retain their carbohydrate-binding properties, we conducted a binding study using OL-TAT and asialofetuin (ASF) as model systems, Fig. 2. OL-TAT was selected because it incorporates the well-studied TAT sequence, which contains multiple Arg and Lys residues, while ASF was chosen as a well-characterized glycoprotein bearing three tri-antennary *N*-linked oligosaccharides with terminal Gal residues and three O-linked Galβ1–3GalNAc disaccharides.^29–30, 53^ These monosaccharide moieties are accessible for binding by OL-TAT peptide. Binding affinities of OL-TAT toward ASF were determined by ITC. As shown in Figure X, OL-TAT binds ASF with a *K*_d_ of 32.5 μM, whereas the TAT control peptide binds ASF with a *K*_d_ of 51.6 μM. The observed slightly weaker TAT binding to ASF could potentially be explained by nonspecific electrostatic interactions. The *K*_d_ value for OL-TAT is comparable to our previously reported affinities for OL/ASF *interactions*.^20–21^ The ITC results indicate that incorporation of a positively charged sequence at the OL *N*-terminus does not diminish its ability to bind ASF, suggesting the possibility that OL-TAT can interact with carbohydrate moieties expressed on the olfactory epithelium, potentially enhancing its residence time in the nasal cavity and thereby increasing its uptake into the brain.

In summary, we have synthesized a series of OL-based peptides incorporating positively charged sequences at the *N*-terminus and demonstrated their dual RNA and carbohydrate-binding properties. Positioning the cationic sequence at the N-terminal end minimizes potential steric interference between the two functional domains, thereby preserving the intrinsic carbohydrate-binding activity of the OL motif while enabling robust RNA interaction. Peptides containing a greater number of positively charged residues exhibited the strongest RNA binding, as confirmed by EMSA and thermal-stability analyses of peptide/RNA complexes using CD spectroscopy. Our binding studies further indicate that the OL ring contributes additional stabilization to RNA/peptide complexes, with a more pronounced effect in peptides that are otherwise weaker RNA binders. Importantly, incorporation of the positively charged sequence does not impair the carbohydrate-binding function of the OL derivatives, as demonstrated by ITC experiments using OL-TAT and ASF as a model system. Collectively, our results justify further development of OL-CPP as bifunctional ligands with tunable RNA and carbohydrate recognition, enabling new intranasal RNA-delivery strategies for targeted transport to the brain.

## Supplementary Material

This is a list of supplementary files associated with this preprint. Click to download.

Onlinefloatimage1.png

PCudicSupplementaryInformationSIPCedits.pdf

Scheme 1 is available in the Supplementary Files section.

## Figures and Tables

**Table 1:** Melting temperatures (T_m_) of peptide/RNA complexes determined by CD spectroscopy. Measurements were performed from 10°C to 90°C at 260 nm, with values averaged over three independent replicates. The RNA concentration was 1.1 μM in all measurements, while peptide concentrations were adjusted to achieve approximately complete RNA saturation as determined by EMSA. Errors represent the standard deviation of the calculated T_m_ value.

RNA	T_m_ (^°^C)
	31.23 (+/− 1.95)
OL	38.36 (+/− 1.16)
OL-(Arg)_12_	43.17 (+/− 0.87)
(Arg)_12_	41.82 (+/− 1.40)
OL-(Arg)8	40.93 (+/− 2.24)
(Arg)_8_	39.79 (+/− 0.56)
OL-TAT	37.77 (+/− 0.22)
TAT	34.40 (+/− 0.16)
OL-(Arg)_4_	36.53 (+/− 1.30)
(Arg)_4_	38.77 (+/− 1.95)
